# Overview of immune checkpoint inhibitor associated myocarditis mechanisms diagnostics and treatment

**DOI:** 10.3389/fimmu.2025.1677984

**Published:** 2025-11-28

**Authors:** Shuhui Feng, Bingru Zhao, Shuo Sha, Xingpeng Bu, Zhenzhen Zhang, Guoying Liu, Huanzhen Chen

**Affiliations:** 1 Department of Cardiology, First Hospital of Shanxi Medical University, School of Medicine, Shanxi Medical University, Taiyuan, China; 2 The First Clinical Medical School of Shanxi Medical University, Taiyuan, China; 3 Department of Geriatrics, Bethune Hospital, School of Medicine, Shanxi Medical University, Taiyuan, China; 4 Department of Emergency, First Hospital of Shanxi Medical University, School of Medicine, Shanxi Medical University, Taiyuan, China

**Keywords:** immune checkpoint inhibitors, myocarditis, immune responses, electrocardiography, cardiovascular magnetic resonance, therapy

## Abstract

Immune checkpoint inhibitor–associated myocarditis (ICI-M) has emerged as a rare yet fulminant immune-related adverse event, characterized by high mortality and diagnostic complexity. Recent studies implicate loss of immune tolerance through PD-1/PD-L1 or CTLA-4 blockade, expansion of autoreactive CD8^+^ T cells, cross-reactivity between tumor and cardiac antigens, and downstream inflammatory cascades as central drivers of myocardial injury. Oxidative stress, endothelial activation, and fibrotic remodeling further amplify damage. Clinically, ICI-M presents with heterogeneous symptoms ranging from subtle conduction abnormalities to fulminant cardiogenic shock. While cardiac troponins and electrocardiography offer early screening, advanced imaging—particularly cardiovascular magnetic resonance with updated Lake Louise Criteria and strain-based analysis—enables more sensitive detection. This review summarizes current insights into the immunopathogenesis, diagnostic approaches, and emerging therapeutic strategies for immune checkpoint inhibitor–associated myocarditis, highlighting the roles of autoreactive T cells, shared tumor–cardiac antigens, advanced imaging, and immunosuppressive interventions in mitigating its high morbidity and mortality.

## 1 Introduction

Immune checkpoint inhibitors (ICIs), a class of monoclonal antibodies (mAbs) specifically targeting immune checkpoints and their ligands, have emerged as transformative agents capable of reversing T cell suppression induced by malignant cells. Clinically approved ICIs—such as cytotoxic T lymphocyte-associated antigen 4 (CTLA-4), programmed cell death protein 1 (PD-1), its ligand PD-L1, and lymphocyte activation gene-3 (LAG-3)—have significantly improved outcomes in various malignancies by enhancing antitumor immune responses. In recent years, the clinical utility of ICIs in cancer therapy has expanded considerably; however, their growing use has been accompanied by an increasing incidence of immune-related adverse events (irAEs), which may affect multiple organ systems, including the lungs, liver, gastrointestinal tract, skin, and, more recently, the cardiovascular system (1). Among these, ICI-associated myocarditis has gained particular attention due to its clinical implications.

ICI-induced myocarditis (ICI-M) often presents with non-specific manifestations and may coexist with arrhythmias, myositis, pneumonitis, or heart failure. Common symptoms include dyspnea, palpitations, peripheral edema, nausea, and fatigue (2, 3). Although the reported incidence remains relatively low, the condition is associated with a high mortality rate, reaching up to 50% (4). Therefore, timely and accurate identification of ICI-associated myocarditis is of paramount importance. Nevertheless, the diagnosis remains challenging due to the absence of pathognomonic clinical features, limited understanding of the underlying mechanisms, and lack of standardized diagnostic criteria. These factors likely contribute to the underestimation of its true incidence. In light of these challenges, this review summarizes current insights into the pathogenesis, diagnostic modalities, and therapeutic strategies for ICI-associated myocarditis, aiming to enhance clinical recognition and management of this potentially fatal complication.

## 2 Potential pathogenic mechanisms of ICI-M

### 2.1 T cell–mediated immune responses

**Figure 1:**
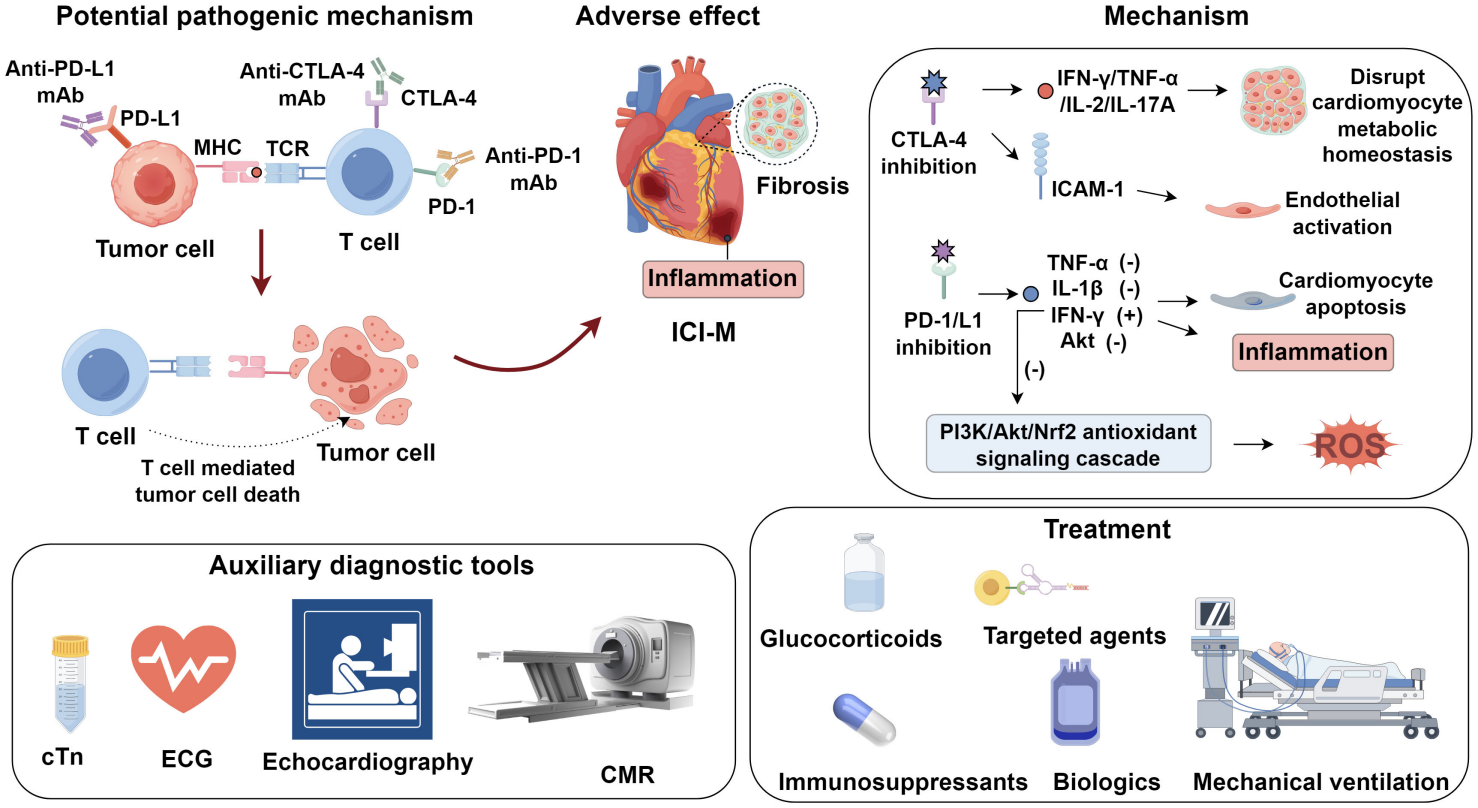
Immune checkpoint inhibitor-associated myocarditis.

Mounting evidence indicates that T cell–mediated immune activation plays a pivotal role in the pathogenesis of ICI-M (5). Under physiological conditions, immune checkpoints CTLA−4 and PD−1 restrain T−cell activation to preserve self−tolerance. CTLA−4 competes with CD28 for CD80/CD86 on antigen−presenting cells, attenuating early T−cell priming, whereas PD−1 engagement with PD−L1/PD−L2 recruits SHP−2 phosphatase to inhibit proliferation, cytokine release, and cytotoxicity in peripheral tissues (6, 7). Checkpoint blockade disrupts this equilibrium, and dual inhibition provokes unrestrained expansion of autoreactive clones and hyperactive cytotoxic T lymphocytes, culminating in immune−related myocarditis (8). In ICI−M, activated CD8^+^ T cells mediate cardiomyocyte apoptosis via perforin− and granzyme B–dependent caspase activation and Fas–FasL interactions triggering death−receptor signaling—pathways recognized as central to myocardial injury in ICI−M (9, 10). Histopathological analyses of myocardium from ICI-M patients have revealed substantial infiltration of T lymphocytes (5). Notably, genetic ablation of CTLA-4 or PD-1 in murine models results in spontaneous autoimmune myocarditis or cardiomyopathy with high lethality (11). Mechanistic studies further implicate autoreactive T cells targeting cardiac α-myosin heavy chain (α-MyHC), a contractile protein uniquely expressed in cardiac muscle, as central mediators in ICI-M. In preclinical models, immunization with α-MyHC peptides induces autoimmune myocarditis, suggesting its potent antigenicity (12). T cell receptor (TCR) repertoires expanded in mice with fulminant myocarditis exhibit clonotypes shared with both cardiomyocytes and skeletal muscle cells from ICI-M patients (13), indicating that α-MyHC-reactive T cells may arise due to cross-priming against shared epitopes. Recent analyses of TCR sequencing in clinical specimens further support the antigen mimicry hypothesis: identical or highly similar TCR clonotypes were identified in both tumor tissues and inflamed myocardium from the same patients who developed ICI-M. This TCR overlap suggests that activated cytotoxic T cells targeting tumor antigens may also recognize structurally homologous cardiac self-antigens such as α-MyHC, thereby facilitating off-tumor immune cross-reactivity (3). Depletion of CD8^+^ T cells significantly enhances survival in Pdcd1^-^/^-^Ctla4^+^/^-^ mice, while adoptive transfer of immune cells from ICI-M donor mice into recipients (without CD8^+^ T cell depletion) induces fatal myocarditis, underscoring the indispensable role of CD8^+^ T cells in disease development (13) (**Figure 1**).

### 2.2 Cardiac antigen release and immune amplification

A mechanistic link has been proposed wherein shared antigens between tumor cells and cardiomyocytes drive T cell–mediated autoimmunity. Johnson et al. (14) demonstrated that, in patients who developed autoimmune myocarditis following ICI therapy, tumor-reactive T lymphocytes also infiltrated cardiac and skeletal muscle tissues. These findings suggest that the therapeutic activation of cytotoxic T cells, while targeting neoplastic lesions, may elicit off-tumor immune recognition against structurally homologous cardiac antigens, thereby precipitating myocarditis and myositis. In addition to direct T cell–mediated cytotoxicity, subclinical myocardial injury has been implicated as a priming factor for ICI-M (3, 15). Surgical resection and chemoradiotherapy, common components of oncologic treatment, can cause structural damage to cardiomyocytes, resulting in the release of cardiac-specific autoantigens into the circulation (16, 17). These antigens may subsequently be recognized by autoreactive B cells, driving the production of pathogenic autoantibodies. The engagement of these autoantibodies with exposed myocardial antigens can initiate antibody-dependent cellular cytotoxicity, culminating in myocardial necrosis and inflammation (18). Moreover, cardiomyocyte injury leads to the release of damage-associated molecular patterns (DAMPs), which act as endogenous danger signals. These DAMPs can potentiate both innate and adaptive immune responses, further amplifying cardiac inflammation (19–21). Notably, some DAMPs may share structural motifs with non-cardiac antigens or exhibit cross-reactivity, thereby broadening the autoimmune response and exacerbating tissue damage (22, 23). This multilayered cascade underscores the complexity of ICI-M pathogenesis, involving not only T cell–mediated cytotoxicity but also B cell–driven humoral mechanisms and innate immune amplification.

### 2.3 Pro-inflammatory and redox mechanisms

Preclinical models have shown that dual blockade of PD-1 and CTLA-4 induces a pro-inflammatory phenotype in cardiac tissues through upregulation of NF-κB, NLRP3 inflammasome components, and myeloid differentiation primary response 88 (MyD88) (24). CTLA-4 inhibition augments levels of pro-inflammatory cytokines such as IFN-γ, TNF-α, IL-2, and IL-17A, which disrupt cardiomyocyte metabolic homeostasis and correlate with increased mortality (25). Moreover, CTLA-4 blockade enhances endothelial activation by upregulating ICAM-1 expression in the aortic endothelium. ICAM-1 silencing has been shown to ameliorate cardiac inflammation and improve contractile function (26). PD-1/PD-L1 inhibitors further potentiate inflammation via induction of cytokines including TNF-α and IL-1β, augment IFN-γ expression, and downregulate phosphorylated Akt, thereby promoting cardiomyocyte apoptosis and inflammatory injury (27). Cardiac fibrosis represents another pathological consequence of immune checkpoint inhibition. Quagliariello et al. (24) reported that CTLA-4 inhibitors regulate the expression of profibrotic mediators such as galectin-3, procollagen-1α, and MMP-9, thereby driving fibrotic remodeling. In patients with low PD-1 expression, Zhang et al. (28) observed elevated TGF-β1, a central cytokine in fibrogenesis. PD-1 blockade also enhances the expression of profibrotic factors within cardiomyocytes, further contributing to fibrotic pathology (24). Oxidative stress further contributes to MMP-9 activation via redox-sensitive cascades, including NF-κB and p38 MAPK, leading to ECM degradation (collagen, laminin), compromised myocardial integrity, and interstitial fibrosis (29). Sustained MMP-9 overexpression under oxidative conditions perpetuates maladaptive ECM turnover, promotes ventricular dilation, and contributes to chronic systolic dysfunction in ICI-M. Activation of T lymphocytes in the context of ICI therapy can induce the generation of reactive oxygen species (ROS), culminating in oxidative stress. Aboelella et al. (30) established a causal relationship between PD-1/PD-L1 blockade and ICI-related cardiotoxicity through ROS-mediated mechanisms. Elevated IFN-γ^+^ macrophages were found to mediate PD-1 inhibitor–induced oxidative injury (31). IFN-γ contributes to ROS generation by suppressing the PI3K/Akt/Nrf2 antioxidant signaling cascade, thereby diminishing cellular antioxidant capacity (32).

## 3 Auxiliary diagnostic tools for ICI-M

### 3.1 Cardiac troponins

cTn represents the most clinically validated biomarkers for diagnosing myocarditis. Approximately 94% of myocarditis patients exhibit elevated serum cTn levels, underscoring the diagnostic sensitivity of this marker (33). In the setting of ICI-M, serum cardiac troponin T (cTnT) concentrations often increase markedly, frequently surpassing 4,000 ng/L (14). Comparative analyses have suggested that cardiac troponin I (cTnI) may offer superior diagnostic specificity over cTnT in the context of ICI-M (34). Further evidence indicates that baseline high-sensitivity troponin T (hs-TnT) levels can effectively predict cardiovascular composite endpoints and myocardial involvement within three months following ICI therapy, with an optimal threshold of 14 ng/L (35).The utility of cTn as an organ-specific biomarker capable of detecting early myocardial injury and potentially forecasting subsequent cardiac dysfunction (36). Nevertheless, concerns regarding isoform specificity persist. For instance, cTnT may be aberrantly expressed in regenerating skeletal muscle, and cTnI autoantibodies are commonly detected in inflammatory diseases or non–ICI-related cardiomyopathies (37). These limitations highlight the need for further comparative studies to identify the most reliable troponin isoform for ICI-M diagnosis. Beyond cTn, several additional biomarkers have been explored for their auxiliary diagnostic value. Elevations in creatine kinase (CK) and its CK-MB are often observed, although both exhibit limited sensitivity and specificity. In certain cases, CK elevation may even precede troponin increases, suggesting a potential early marker of myocardial involvement (38, 39). Notably, 88% of ICI-M patients present with elevated N-terminal pro–brain natriuretic peptide (NT-proBNP), a surrogate of myocardial stress (40). Moreover, circulating levels of ANGPTL2 have been found to be increased in ICI-M, indicating a novel diagnostic candidate that may enhance accuracy when used in combination with established cardiac biomarkers (41) (**Table 1**).

**Table 1:** Summary of cardiac biomarkers in ICI-associated myocarditis.

Category	Biomarker	Pathophysiological role	Diagnostic/prognostic utility	Limitations
Myocardial injury	cTn T/ cTn I	Structural proteins released during cardiomyocyte necrosis	Early and sensitive marker; correlates with disease severity	Possible cross-reactivity with skeletal muscle; autoantibody interference
Myocardial injury	CK, CK-MB	Enzymes released from damaged myocytes	Useful adjunct when cTn unavailable; may precede cTn rise	Low specificity; influenced by skeletal muscle injury
Hemodynamic stress	NT-proBNP	Released due to ventricular wall stress and stretching	Reflects hemodynamic burden; prognostic for cardiac dysfunction	Elevated in renal failure or sepsis; nonspecific
Novel marker	ANGPTL2	Inflammatory cytokine linked to endothelial dysfunction	Potential adjunct biomarker for ICI-M diagnosis	Limited validation; small patient cohorts

### 3.2 Immune-mediated electrophysiological disruption in ICI-associated myocarditis

Electrocardiography remains a widely accessible and cost-effective modality for the initial evaluation of cardiac function. Emerging evidence underscores its utility in ICI-M, a condition frequently marked by heterogeneous electrocardiographic manifestations, particularly arrhythmias (33, 42, 43). In a multicenter retrospective study, ICI-M was found to be significantly associated with the emergence of novel conduction disturbances, including bundle branch blocks and complete atrioventricular block, alongside characteristic features such as voltage attenuation and repolarization abnormalities (44). Expanding on this, Song et al. (45) provided a comprehensive characterization of ECG abnormalities in ICI-M, encompassing sinus arrhythmia, ventricular arrhythmias, atrial fibrillation, and atrial flutter. These findings underscore the critical importance of vigilant ECG monitoring for the early detection and effective management of ICI-M–related cardiotoxicity. These conduction abnormalities arise from cardiac inflammation, which promotes arrhythmogenic events through several interrelated mechanisms. Dynamic interactions between infiltrating immune cells and resident cardiac fibroblasts or cardiomyocytes drive fibrotic remodeling, thereby disrupting normal electrical propagation (46, 47). Moreover, increasing evidence suggests that immune cells—particularly macrophages—may directly modulate electrical conduction within the myocardium. In addition, inflammatory mediators such as autoantibodies and cytokines have been shown to alter ion channel function in cardiomyocytes, further contributing to electrical instability (48, 49). Notably, conduction disturbances are also prevalent among individuals with systemic inflammatory disorders, suggesting that even extra-cardiac inflammation can exert remote effects on myocardial electrophysiology (45, 50). Prolongation of QRS duration has emerged as a prognostic marker in ICI-M, with a QRS interval ≥110 ms conferring elevated risk of major adverse cardiovascular events (MACE), thereby underscoring its potential utility in clinical risk stratification (50). Nevertheless, the overall incidence of arrhythmias among patients with ICI-M remains relatively low (51), suggesting that while QRS prolongation may indicate heightened risk, it is not universally observed. Accordingly, although ECG findings are not pathognomonic for ICI-M, electrocardiography remains an essential adjunctive tool for early detection and longitudinal assessment of cardiotoxicity in this patient population.

### 3.3 Imaging modalities

#### 3.3.1 Echocardiography

Echocardiography allows comprehensive assessment of cardiac morphology and function, including left ventricular ejection fraction (LVEF) and myocardial strain indices. While LVEF reduction is a hallmark of cardiotoxicity (52), cardiotoxicity may also occur in patients with preserved LVEF (53). Awadalla et al. first used global longitudinal strain (GLS) to detect ICI-M, showing significantly reduced GLS, correlating with troponin but not LVEF (40). Quinaglia et al. similarly observed GLS impairment regardless of LVEF status (54). Tanabe et al. found that preserved-LVEF patients with GLS <16% had higher major adverse cardiovascular events (MACE) incidence versus GLS ≥16% (55), indicating GLS’s superior sensitivity over LVEF. Beyond GLS, global circumferential strain (GCS) and radial strain (GRS) also decline in ICI-myocarditis, offering prognostic value (54). Early-stage disease may present with normal chamber dimensions, limiting conventional echocardiography’s diagnostic utility (56). Two-dimensional speckle tracking echocardiography (2DSTE), widely used clinically, provides high spatial resolution, LVEF-independent analysis, and noise resistance but is affected by temporal resolution, afterload, and image quality (57, 58). Conversely, three-dimensional speckle tracking echocardiography (3DSTE) improves reproducibility, enables holistic left ventricular assessment, and includes parameters like area strain. However, 3DSTE requires optimal acoustic windows, high-quality datasets, and patient cooperation (breath-holding, stable heart rate) (59), with strain metrics varying by ultrasound platform (59). Regardless of method, echocardiographic strain imaging is a valuable adjunct for diagnosing ICI-associated myocarditis.

#### 3.3.2 Cardiovascular magnetic resonance

CMR, offering superior soft-tissue contrast and spatial resolution, is now the preferred modality for diagnosing immune ICI-M (52, 60). The Lake Louise Criteria (LLC), a widely adopted CMR-based framework, initially evaluated myocardial edema, hemorrhage, fibrosis, and perfusion anomalies (56). The revised LLC now integrates T1/T2 mapping and extracellular volume (ECV) measurements, which markedly enhance diagnostic sensitivity and specificity in ICI-M, surpassing the original framework (60–63). Furthermore, advanced CMR modalities—such as feature-tracking strain analysis and texture-based radiomics—enable nuanced assessment of myocardial deformation and tissue heterogeneity (58). Quantitative T1 and T2 mapping overcome the limited sensitivity of conventional weighted imaging, providing superior detection of inflammatory edema and fibrotic changes (64–66). In ICI-myocarditis cohorts, elevated T1 and T2 values were common, with baseline T1 independently predicting major adverse cardiac events (67). Notably, T2 is more specific for edema, whereas T1 may reflect both inflammation and fibrosis (64). Late gadolinium enhancement (LGE), indicative of myocardial injury, is detected in only 48% of ICI-M cases and is more prevalent among patients with reduced rather than preserved LVEF (68). Septal LGE, though less frequent than in viral myocarditis, correlates with increased MACE risk (46). Early-phase ICI-myocarditis may lack LGE due to absent fibrosis, but LGE extent associates with CD8^+^ T-cell infiltration, suggesting diagnostic utility (69, 70). Feature-tracking strain analysis mitigates the operator dependency inherent in echocardiography (71), offering high resolution and efficiency (72–74). In ICI-myocarditis, impaired LV global longitudinal strain indicates subclinical dysfunction despite preserved LVEF (75–77). Combining LLC with left atrial strain (LA SRe) and LV GLS improves diagnostic performance (77). CMR radiomics, including texture analysis, detects subtle tissue heterogeneity, aiding in infarction and myocarditis diagnosis (78–80). Though unexplored in ICI-myocarditis, its sensitivity to early tissue changes holds promise (81). EMB remains the gold standard for diagnosing ICI-associated myocarditis (58). Few studies describe immune infiltrates, which typically include CD8^+^ and CD4^+^ T lymphocytes, as well as CD68^+^ macrophages (5, 14). Given its invasiveness, EMB is typically reserved for diagnostically ambiguous cases where non-invasive imaging is inconclusive. CD68^+^ macrophages localize perivascularly and interstitially, secreting TNF-α and IL-1β (82). Emerging data suggest M1-polarized macrophage responses exacerbate myocardial damage. These signatures imply synergistic immune cytotoxicity by T cells and macrophages (83, 84).

## 4 Treatments of ICI-M

### 4.1 First-line therapy

The therapeutic approach to ICI-M primarily involves discontinuation of ICIs, administration of glucocorticoids, and symptomatic supportive care (85). Given the fulminant course and poor prognosis of severe ICI-M, aggressive immunosuppressive therapy should be promptly instituted. When feasible, adjunctive interventions such as plasma exchange and advanced life support should be considered. Glucocorticoids remain the cornerstone of first-line therapy for ICI-M. However, consensus regarding the optimal initiation dose, timing, tapering regimen, and duration of therapy remains lacking. Evidence suggests that early administration (within 24 hours of onset) of high-dose corticosteroids is associated with improved clinical outcomes (86). Upon diagnosis of fulminant or non-fulminant ICI-M, immediate initiation of intravenous methylprednisolone is recommended to mitigate the risk of major adverse cardiac events (MACE). If clinical improvement is observed—indicated by a reduction in peak cTn levels by >50% within 24–72 hours, alongside resolution of left ventricular dysfunction, atrioventricular block, or other arrhythmias—a transition to oral prednisone is advised (87). Although an optimal tapering schedule has yet to be established, dose reduction can be guided by clinical symptoms, cTn levels, and electrocardiographic changes. A common approach involves weekly dose reductions of prednisone by 10 mg. When the dose reaches 20 mg/day, re-evaluation of left ventricular function and cTn levels is necessary. If stable, subsequent dose reductions can proceed at 5 mg/week until 5 mg/day, followed by 1 mg/week thereafter.

### 4.2 Second-line therapy

In glucocorticoid-refractory ICI-M—defined as ≤50% reduction in peak cardiac troponin after 3 days of intravenous methylprednisolone and cardiac support, or persistent arrhythmias, AV block, or ventricular dysfunction—escalation to second-line immunosuppressants is warranted. Therapeutic options include chemotherapeutics (mycophenolate mofetil), targeted agents (tofacitinib), and biologics such as anti-thymocyte globulin, alemtuzumab, tocilizumab, abatacept, and IVIG. Salem et al. (88) reported that combining the JAK2 inhibitor ruxolitinib with abatacept, alongside monitoring CD86 receptor occupancy, reduced ICI-M mortality to 3% in patients requiring mechanical ventilation. Complement inhibition with eculizumab may benefit patients presenting with overlapping myasthenia-myositis-myocarditis syndromes (89). Immunosuppressive regimens must be tailored to clinical context and practitioner expertise. In fulminant cases unresponsive to second-line therapy and complicated by hemodynamic collapse, prompt ICU admission is essential. Mechanical circulatory support should be initiated early. In patients with hypotension and early shock (tachycardia), micro-axial flow pump insertion is advised (90). Besides, plasma exchange was recommended to be used as early as possible and as part of multimodal therapy (91). This multi-modal strategy underscores the urgency of rapid recognition and individualized escalation in ICI-M management.

## 5 Conclusion

ICI-associated myocarditis represents a severe immune-related adverse event with complex pathophysiology and high mortality. Current understanding highlights the central role of T cell–mediated autoimmunity, molecular mimicry, and inflammatory cascades in driving myocardial injury. While troponins and ECG provide initial diagnostic clues, advanced imaging—particularly CMR with T1/T2 mapping and strain analysis—has become indispensable for early detection and risk stratification. EMB, though definitive, is limited by invasiveness and should be reserved for diagnostically uncertain cases. Treatment hinges on prompt immunosuppression, with high-dose glucocorticoids as first-line therapy and emerging biologics showing promise in refractory disease.

Nevertheless, significant knowledge gaps remain. First, there is a lack of validated biomarkers that can reliably predict ICI-associated myocarditis prior to clinical manifestation. Second, multicenter prospective registries are urgently needed to refine diagnostic criteria and capture the full clinical spectrum of this disease across diverse populations. Third, the absence of standardized immunosuppressive protocols highlights the need for well-designed randomized controlled trials to guide the selection, timing, and tapering of therapeutic agents. Addressing these challenges will require concerted multidisciplinary efforts, combining oncological, cardiological, and immunological expertise, to improve diagnostic accuracy, therapeutic precision, and ultimately, patient outcomes in this high-risk population.
